# The Ghent Psychotherapy Study (GPS) on the differential efficacy of supportive-expressive and cognitive behavioral interventions in dependent and self-critical depressive patients: study protocol for a randomized controlled trial

**DOI:** 10.1186/s13063-017-1867-x

**Published:** 2017-03-14

**Authors:** Reitske Meganck, Mattias Desmet, Claudi Bockting, Ruth Inslegers, Femke Truijens, Melissa De Smet, Rosa De Geest, Kimberly Van Nieuwenhove, Vicky Hennissen, Goedele Hermans, Tom Loeys, Ufuoma Angelica Norman, Chris Baeken, Stijn Vanheule

**Affiliations:** 1 Department of Psychoanalysis and Clinical Consulting, Faculty of Psychology and Educational Sciences, Ghent University, Henri Dunantlaan 2, Ghent, 9000 Belgium; 2 Department of Clinical Psychology, Social and Behavioral Sciences, Universiteit Utrecht, Heidelberglaan 1, Utrecht, 3584 CS Netherlands; 3 Department of Data Analysis, Faculty of Psychology and Educational Sciences, Ghent University, Henri Dunantlaan 1, Ghent, 9000 Belgium; 4 Department of Psychiatry and Medical Psychology, Faculty of Medicine and Health Sciences, Ghent University, De Pintelaan 185, K12, Ghent, 9000 Belgium

**Keywords:** Major depressive disorder, Cognitive behavioral therapy, Short-term psychodynamic psychotherapy, Personality styles

## Abstract

**Background:**

Major depressive disorder is a leading cause of disease burden worldwide, indicating the importance of effective therapies. Outcome studies have shown overall efficacy of different types of psychotherapy across groups, yet large variability within groups. Although patient characteristics are considered crucial in understanding outcome, they have received limited research attention. This trial aims at investigating the interaction between therapeutic approach (pre-structured versus explorative) and the personality style of patients (dependent versus self-critical), which is considered a core underlying dimension of depressive pathology.

**Methods/design:**

This study is a pragmatic stratified (dependent and self-critical patients) parallel trial with equal randomization (allocation 1:1) conducted in Flanders, Belgium. One hundred and four patients will be recruited and randomized to either 16–20 sessions of cognitive behavioral therapy for depression (pre-structured approach) or 16–20 sessions of short-term psychodynamic psychotherapy for depression (explorative approach) conducted by trained psychotherapists in private practices. The primary outcome is the severity of depression as measured by the Hamilton Rating Scale for Depression at completion of therapy. Secondary outcome measures include self-reported depressive and other symptoms, interpersonal functioning, idiosyncratic complaints, and the presence of the diagnosis of depression. Additional measures include biological measures, narrative material (sessions, interviews), and health care costs.

**Discussion:**

This trial presents the test of an often-described, yet hardly investigated interaction between important personality dimensions and therapeutic approach in the treatment of depression. Results could inform therapists on how to match psychotherapeutic treatments to specific personality characteristics of their patients.

**Trial registration:**

Isrctn.com, ISRCTN17130982. Registered on 2 February 2015.

**Electronic supplementary material:**

The online version of this article (doi:10.1186/s13063-017-1867-x) contains supplementary material, which is available to authorized users.

## Background

Major depressive disorder (MDD) is the leading cause of disability worldwide and a major contributor to the overall global burden of disease [1]. This implies that further development of effective therapies is essential for public health. Therapies for MDD promoted as evidence-based are antidepressant medication and different types of psychotherapy, including cognitive behavioral therapy (CBT) and psychodynamic therapy (PDT) [2, 3]. While outcome studies demonstrate overall efficacy of these therapies *across groups*, they also reveal substantial variability *within groups* between individual patients [4]. Consequently, identifying patient-treatment interaction effects in outcome research is a major challenge in optimizing the efficiency of psychotherapy.

There is preliminary evidence suggesting that the patient’s global personality structure in terms of *dependency* and *self-criticism* predicts differential treatment response to the basic nature of the therapy in terms of being *directive* or *explorative*. A vast amount of empirical research from both psychodynamic [5] and cognitive behavioral [6] points of view identified two personality traits — dependency and self-criticism — that both render a person vulnerable to MDD [7]. Persons with predominantly dependent personalities are characterized by interpersonal dependency and strong wishes to be loved and protected. Depressive complaints are marked by feelings of helplessness, weakness, and intense fears of being abandoned. Persons with a predominantly self-critical personality on the other hand are more focused on achievement and living up to their own high standards and expectations. Depressive complaints are more related to the experience of failure to live up to these standards and feelings of inferiority and guilt [5, 6, 8]. It was demonstrated that, depending on the dominant underlying personality traits, patients with MDD are susceptible to specific life stressors and show distinct depressive symptom patterns [9, 10]. Furthermore, recent naturalistic outcome research [4, 7, 11–13] suggests that dependent and self-critical MDD patients respond differently to directive versus explorative therapies. Directive therapies such as cognitive behavioral therapy, as the most studied directive approach, are more “structured, present-oriented psychotherapies directed toward solving current problems and teaching clients skills to modify dysfunctional thinking and behavior [14]”. In explorative approaches, such as most psychodynamically oriented psychotherapies, therapeutic interventions are tuned to the spontaneous way in which patients present new material (for example narrative descriptions of thoughts, emotions, and complaints) and their broader (interpersonal and historical) context. Although explorative approaches withhold from directive interventions, they are compatible with the use of a manual that prescribes the set of theoretical principles and therapeutic techniques that are used during the explorations [15, 16].

Post hoc analyses suggest that different mediators of change are at work in dependent and self-critical patients. Directive interventions seem to alleviate depressive symptoms in dependent patients because the structure and support positively affect the *interpersonal functioning* of the patients; explorative interventions appear to alleviate depressive symptoms in self-critical patients because they promote *intrapersonal insight* [4, 7, 10, 12, 13]. Further post hoc analysis [4] even suggested that explorative approaches might *inhibit* therapeutic progress in dependent patients, because they experience the lack of directedness as a lack of support. Similarly, directive approaches might *inhibit* progress in self-critical patients, because they experience the therapist’s directedness and structure as coercive [4, 7, 10, 12, 13].

With the increasing emphasis on directive and structured treatment approaches, it might be of major clinical importance to explore the way more directive and more open treatment styles suit different personality styles. In existing research on differential efficacy of interventions in dependent and self-critical patients, researchers always re-analyzed data from previously executed psychotherapy research (see descriptions above). Judges first distinguished between dependent and self-critical MDD patients on the basis of pre-treatment case formulations and subsequently studied in which types of therapy the highest efficacy was achieved [4, 10]. Post hoc methodology is a valuable tool for generating new ideas; however, it lacks internal consistency to yield a firm evidential basis [17]. Rather, this topic requires a design in which patients are assigned to a controlled experimental treatment procedure.

## Methods/design

### Study aims

The primary aim is to present an experimental test of the main hypothesis deduced from the post hoc observations discussed above concerning differential efficacy of directive (CBT) and explorative treatment (PDT) for different personality types. In this trial, the central hypothesis that is tested is that there is a significant interaction effect between type of patient (dependent versus self-critical) and type of therapy (directive versus explorative) in predicting outcome. More specifically, we expect that directive treatment will yield significantly better outcome in terms of observer-rated depression severity in dependent compared to self-critical patients, while explorative treatment will yield significantly better outcome in terms of observer-rated depression severity in self-critical compared to dependent patients.

Secondary aims concern secondary outcomes such as percentage of patients in remission and recovery, self-reported depression, symptom severity and well-being, and interpersonal functioning. Furthermore, mediators and processes of change, clinical predictors, patient perspectives on change and helpful elements, impact of research on therapists and patients, biomarkers of depression, and long-term outcomes among others will be explored in a number of consecutive studies following the main trial.

### Study design

**Fig. 1:**
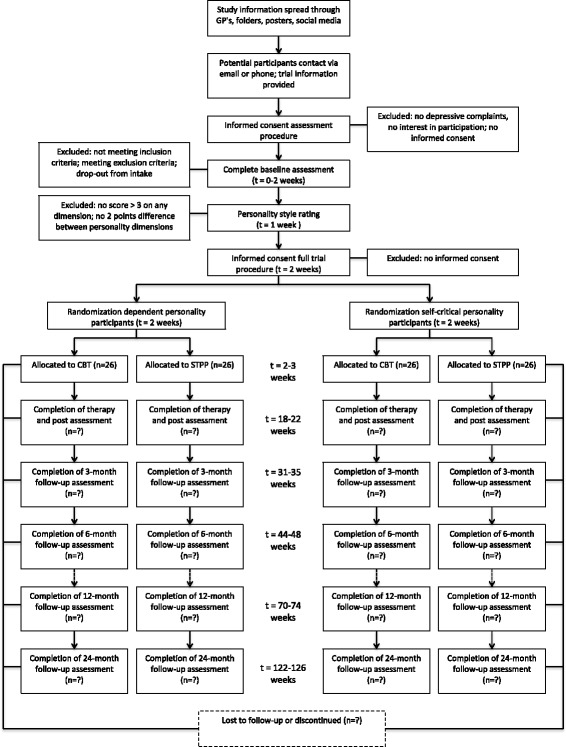
SPIRIT flow diagram of progress through the phases of the Ghent Psychotherapy Study

The study will be a pragmatic stratified (dependent and self-critical patients) parallel trial with equal randomization (allocation ratio 1:1) conducted in Flanders, Belgium. The study compares a predominantly explorative and a predominantly directive intervention, namely short-term psychodynamic psychotherapy for depression (STPP, [15, 18]) and cognitive behavioral therapy for depression (CBT-D, [19, 20]) respectively. The study design as described here adheres to the Standard Protocol Items: Recommendations for Interventional Trials (SPIRIT) guidelines [21, 22], including a SPIRIT flow diagram (Fig. 1), SPIRIT schedule (Table 1), and checklist (Additional file 1).

**Table 1:** SPIRIT schedule of enrollment, interventions, and assessments

	Study period									
	Enrollment	Allocation	Post-allocation					Close-out 1		Close-out 2
Time point	-t1	0	t1	t2	t3	t4	t5	t6	t7	t8
Enrollment										
Eligibility screening	x									
Informed consent	x	x								
Allocation		x								
Interventions										
CBT			x	x	x					
STPP			x	x	x					
Assessment										
- Interviews:										
HRSD	x					x				
SCID I	x					x				
SCID II	x									
CDI	x									
CCI					x	x	x	x	x	x
- Self-report scales:										
BDI-II, SCL-90, OQ-45, IIP-32	x			x	x	x	x	x	x	x
DASS, VAS	x		x	x	x	x	x	x	x	x
Idiosyncratic scale			x	x	x	x	x	x	x	x
WAV				x	x					
DEQ, PSI	x					x		x		x
VTCI, ECR	x					x				
ZIL	x				x	x				
- Biological measures										
Saliva samples	x		x	x	x	x	x	x	x	x
Blood samples	x					x				
Hair samples	x				x	x				
- Therapist measures										
Structured report, VAS			x	x	x					
TRQ, VAS				x	x					

t1: sessions 2–3, 5–7, 9–11, 13–15, 17–19; t2: sessions 1, 12, 16, 20; t3: session 8; t4: post; t5: 3 month follow-up; t6: 6 month follow-up; t7: 12 month follow-up; t8: 24 month follow-up

*HRSD* Hamilton Rating Scale for Depression, *SCID I* Structured Clinical Interview for DSM-IV-TR Axis I Disorders, *SCID II* Structured Clinical Interview for DSM-IV-TR Axis II Disorders, *CDI* Clinical Diagnostic Interview, *CCI* Client Change Interview, *BDI-II* Beck Depression Inventory II, *SCL-90* Symptom Checklist -90, *OQ-45* Outcome Questionnaire-45, *IIP-32* Inventory of Interpersonal Problems-32, *DASS* Depression Anxiety Stress Scales, *VAS* Visual analogue scale, *WAV* Working Alliance Inventory, *DEQ* Depressive Experiences Questionnaire, *PSI* Personal Style Inventory, *VTCI* Short Temperament and Character Inventory, *ECR* Experiences in Close Relationships, *ZIL* Self-rating Inventory for Post Traumatic Stress Disorder, *TRQ* Therapist Relationship Questionnaire

### Participants/eligibility criteria

We will recruit adult patients living in Flanders, Belgium with a depressive disorder who meet the following inclusion criteria:

- Current diagnosis of MDD according to Diagnostic and Statistical Manual of Mental Disorders, 4^th^ Edition (DSM-IV)
- Hamilton Rating Scale for Depression (HRSD, [23]) total score >14
- Age between 18 and 65 years old
- Sufficient knowledge of the Dutch language
- Dominance of either dependent or self-critical personality characteristics (prototype matching procedure [24])

Patients on antidepressant medication can still meet inclusion criteria and can participate in the study if they have been on a stable dose for 4 weeks or more. All medication use and changes will be monitored in detail throughout the study.

We aim for a representative sample (to maximize external validity) by keeping the exclusion criteria to a minimum. Exclusion criteria are any of the following:

- Current diagnosis of psychosis, delusions or bipolar disorder
- Acute suicidal risk
- Primary diagnosis of substance abuse/dependence
- Evidence of at least one clinically significant medical condition (e.g., brain damage, degenerative neurological condition) causing cognitive or physical impairments that might prevent full participation in the treatments
- Participation in another ongoing psychotherapeutic treatment
- Explicit preference for a specific type of therapy or a male or female therapist (which implies participants cannot consent to the procedure of random allocation to treatment)

### Recruitment and baseline assessment

The participant flow throughout the study is shown in Fig. 1.

Participants are recruited both by means of referrals from general practitioners and mental health care centers, and by self-referral. Recruitment information is spread via posters, folders, local media, and online publications (social media, etc.). All recruitment information is focused on potential participants who experience depressive complaints and have a voluntary motivation to start psychotherapeutic treatment. Participants apply for the study via email or telephone. After application, an initial phone screening takes place in which the patient is informed about the study procedure and relevant inclusion and exclusion criteria. When there are indications of depressive complaints and the possible participant consents to the research intake procedure, baseline assessment starts.

The baseline assessment takes place at the Faculty of Psychology and Educational Sciences of Ghent University and is conducted by a team of postgraduate research assistants trained in the respective procedures. Before the first intake interview, patients receive a baseline battery of questionnaires and provide eight saliva samples (a morning and evening sample for 4 consecutive days) to measure baseline symptom presence and severity, personality characteristics, and stress levels before the first face-to-face contact with the researcher. During the face-to-face assessment, the Clinical Diagnostic Interview (CDI, [25]), the HRSD [23], and the Structured Clinical Interview for DSM-IV Axis I disorders [26] and Axis II disorders [27] are administered in two respective interview appointments with the same researcher. The CDI is a semi-structured interview that assesses both the current clinical complaints and a range of current and lifetime inter- and intrapersonal experiences. This interview is used to rate the dependent and self-critical personality organization by means of prototype matching [24]. The rating takes place after the CDI and before the HRSD and SCID to limit any possible bias by the formal diagnosis. Three independent and trained researchers (interviewer, one postgraduate researcher, and one academic staff researcher) conduct the prototype matching. They each score the interview individually and consequently discuss their scores. To be possibly included in the trial, a score of at least 3 on a scale of 1 to 5 for one of the personality dimensions and a minimum of 2 points difference with the score on the other personality dimension are required. When no agreement can be reached, the difference in consensus scores between dependent and self-critical personality dimensions is less than 2 points, or there is no score of at least 3 on either dimension, patients are excluded from the trial. The HRSD and SCID-I depression module are used to assess severity of depressive symptoms and the diagnosis of MDD, respectively. The SCID-I and II are also used to assess co-morbidity and exclusion criteria. The baseline procedure finally yields the collection of a hair sample and a blood sample. Together with the saliva samples, these form the baseline for the biological component of the study.

When eligible for the study, patients receive further written and oral information about the interventions and the full research procedure and again sign the informed consent form before being randomized into one of the treatment conditions. They also indicate whether they can be contacted again after the study to allow designing additional waves in the study and extending the follow-up period.

Patients who are not eligible for the study (either by not meeting inclusion criteria or meeting exclusion criteria), yet have a request for psychotherapy, are referred to appropriate care.

### Study setting

The study is carried out by the University of Ghent, Faculty of Psychology and Educational Sciences, Department of Psychoanalysis and Clinical Consulting (Prof. Dr. Mattias Desmet, Prof. Dr. Reitske Meganck) in collaboration with the University of Utrecht (Prof. Dr. Claudi Bockting) and Ghent University Hospital (Prof. Dr. Chris Baeken). The intake procedure, peri-, post- and follow-up assessments all take place at Ghent University. The psychotherapeutic treatments take place in private practices in the area of Ghent, a medium-sized city in Flanders, Belgium, and are carried out by clinical psychologists with psychotherapy training who have been additionally trained to conduct specific treatments for the purpose of this study. Prof. Dr. Mattias Desmet from Ghent University is responsible for the training of the psychodynamic therapists. Prof. Dr. Claudi Bockting from the University of Utrecht is responsible for the training of cognitive behavioral therapists. Statistical analyses are supported by independent statisticians from the Department of Data Analysis at Ghent University.

### Interventions

#### Cognitive behavioral therapy (CBT)

The CBT condition consists of 16–20 sessions of CBT for MDD based on the CBT treatment as described by Beck [20]. Specifically, the three-phase protocol elaborated by Bockting and Huibers [19] is implemented. This CBT is structured and teaches clients skills to modify dysfunctional thinking and behavior. The therapeutic approach encompasses a collaborative approach as a basis for a good working alliance. Each therapy session starts with an agenda setting; that is, the therapist helps the client to select the problems they want to discuss. They then focus on cognitions and beliefs that might contribute to the emotional reaction and/or problems. Socratic questioning will be taught to examine potential catastrophic/negative reasoning. Behaviors that have interfered with the ability to solve problems will be identified, and alternative potential ways of coping with these situations will be explored. Usually, in most sessions they develop an action plan for the following week, focused on cognitions and/or behavior. In CBT, clients themselves are taught the skills and tools to deal with day-to-day problems in their lives and to enhance emotion regulation. In this CBT protocol the main focus in the first phase is on behavioral activation interventions (mainly based on the model of Lewinsohn et al. [28]). The second phase introduces cognitive interventions focused on the identification of negative thoughts (in the here and now) and challenging techniques (such as Socratic questioning). The third phase also encompasses identification of dysfunctional beliefs, challenging techniques, and formulating a personal prevention strategy.

The techniques that are used throughout the three phases are directive interventions partly sequenced (especially the cognitive interventions) according to standard CBT techniques [19, 20, 28].

#### Short-term psychodynamic psychotherapy (STPP)

The STPP condition consists of 16–20 sessions and follows the unified psychodynamic protocol for depression (UPP-depression, [18]). The UPP-depression is a principle-based psychodynamic time-limited treatment integrating the most effective disorder-specific treatment components of empirically supported psychodynamic interventions for depression. It is mainly based on the supportive-expressive intervention continuum outlined by Luborsky [15] and Connolly Gibbons and colleagues [16]. It has a modular format comprising seven integrated modules and aims at a flexible application concerning both sequence and dosage in line with the individual patient’s needs.

The first three modules can be mainly situated in the first phase of therapy. They respectively focus on preparing the patient for psychotherapy, i.e., presenting a rationale and familiarizing the patient with the treatment process; motivating the patient and setting treatment goals, including a discussion of possible ambivalence towards treatment and change; and educating and empowering the patient to become an active participant in treatment.

The two main treatment modules comprise the two dominant treatment principles: supportive interventions on the one hand and expressive interventions on the other. Therapists use the techniques in response to the spontaneous material brought up by the patient, showing the explorative rather than directive nature of the treatment approach. Supportive interventions mainly foster a good working alliance. Specific supportive interventions are described [15] that can be used in a differentiated way depending on the amount of support specific patients need. Expressive techniques, on the other hand, focus on identifying, interpreting, and working through unresolved conflicts [15, 18]. The focus is on the central relationship pattern (core conflictual relationship theme [15, 29]) and understanding symptoms within this relational context.

Finally, in the third treatment phase, attention is paid to termination and relapse prevention.

#### Therapists

Four clinical psychologists with 3 to 8 years of clinical experience and postgraduate training in psychodynamic psychotherapy receive 2 days of training according to the UPP-depression [18] based on the Supportive-Expressive Time Limited (SETL) manual for MDD proposed by Luborsky [15]. Four clinical psychologists with 4 to 8 years of clinical experience and postgraduate training in CBT receive 2 days of training in pre-structured CBT according to the cognitive behavioral protocol for depression by Bockting and Huibers [19], based on the manual proposed by Beck et al. [20].

Both groups of therapists receive regular supervision sessions (bi-weekly group supervision of 2 hours) by experienced therapists in the respective disciplines.

All therapists are blind to the research hypotheses and the outcome of the screening measures and interviews (see below). In both conditions, therapy length is fixed at 16 to 20 sessions (45 minutes each).

#### Intervention fidelity

The extent to which the interventions are delivered as intended, or the treatment integrity, is protected and investigated in a number of ways. First, therapists deliver only the specific intervention that aligns with their basic psychotherapy training; i.e., cognitive behavioral therapists deliver only the CBT protocol for depression and psychodynamic therapists deliver only STPP. Furthermore, the training and regular supervision sessions aim at sufficient competence in and adherence to the respective treatment manuals. Each therapist also had a “training case” that is not included in the trial that was closely supervised, so that they could get used to both the manual and the respective research procedures. All sessions were audiotaped and thus allow explicit adherence checks. Specifically, the Comparative Psychotherapy Process Scale [30] will be used to reliably assess characteristics of cognitive behavioral and psychodynamic-interpersonal treatments in the respective treatments. Therapist adherence/fidelity to the specific protocols will also be assessed.

### Outcomes and study measures

#### Procedure

There are measurement points at intake, before the start of therapy, at every session (limited self-report battery), at every fourth session (extended self-report battery), post-treatment, and at follow-up at 3 and 6 months. In the second wave of the study, additional follow-up is planned at 12 and 24 months, which can be extended if research means are available and if participants consent to prolonged follow-up. All interviews and therapy sessions are recorded on audiotape. Therapists write semi-structured reports of every session including an appreciation of their ability to work along the principles of the respective manuals, a description of the content of the session, and self-report scales. An overview of the full procedure and the respective measures that are used is presented in Table 1 according to SPIRIT guidelines [21, 22].

#### Primary outcome

The primary outcome is the post-treatment assessment with the HRSD [23], which is the most widely used interview-based measure in depression studies [31]. The interview assesses depression severity. Outcome HRSD interviews are conducted by trained researchers who are blind to the research hypothesis, blind to the status of the patient (pre- or post-therapy), and thus also blind to treatment group. These researchers also conduct the post-treatment SCID-I interview.

#### Secondary outcomes

Self-report depression severity is measured by means of the Beck Depression Inventory (BDI-II [32]), which consists of 21 items and shows excellent reliability and validity. The presence of a diagnosis of depression (percentage of patients in remission) and other Axis I disorders is assessed post-treatment by means of the SCID-I interview [26] conducted by the researchers who also conduct the post-treatment HRSD interviews and thus are blind to treatment group and treatment status.

Global symptom severity and functioning are repeatedly measured by means of the Symptom Checklist-90 (SCL-90-R [33]), the shortened Depression Anxiety and Stress Scale (DASS [34]), and the Outcome Questionnaire-45 (OQ-45 [35]). Problems in interpersonal functioning are assessed by means of the Inventory of Interpersonal Problems-32 (IIP-32 [36]).

In line with recent recommendations [37], one to five idiosyncratic complaints are mapped during baseline assessment and rated by the participant at each session on a visual analogue scale (VAS) of 0–10.

#### Other measures

In addition to the prototype matching, commonly used measures for personality styles are included in the study. These are the Personal Style Inventory (PSI [38]) and the Depressive Experiences Questionnaire (DEQ [39]). Because of validity issues [9, 40], these are not used to categorize patients in one of the personality styles, but their administration allows further studies on convergence of different approaches to assess personality and compare results with other studies.

A number of additional measures are administered to provide more context to the other (self-report, interviews, and biological) measures, namely the Self-rating Inventory for Post Traumatic Stress Disorder (ZIL [41]), measuring trauma-related symptoms; the revised version of the Experiences in Close Relationships questionnaire (ECR [42]) designed to assess individual differences with respect to attachment-related anxiety and attachment-related avoidance; and the Short Temperament and Character Inventory (VTCI [43]), providing a comprehensive assessment of personality. These are administered a limited number of times (see Table 1). At every session, all participants rate ten emotions on a VAS of 0–10 [44].

To map the therapeutic relationship, which is considered a crucial variable in explaining outcome [45, 46], both therapists and patients fill out the Working Alliance Inventory-Short Revised (WAI-SR [47, 48]) every fourth session. Therapists additionally fill out the Therapist Response Questionnaire (TRQ [49]), and at each session a shortened version representing the seven main dimensions of the TRQ on a VAS of 0–10.

Finally, the semi-structured Client Change Interview (CCI) is administered around the 8th treatment session, at post-treatment, and at 6 and 24 months follow-up [50]. It inquires about experienced change, to what patients ascribe those changes, and the experience of therapy.

#### Biological measures

Biological measures comprise saliva, hair, and blood samples collected at different time points throughout the study (see Table 1). They will be used to assess cortisol levels as an indicator of stress and to explore biomarkers for depression in a broader way.

Saliva samples are a standard and well-validated way to measure hormonal fluctuations. They are administered before the first intake interview, before the first therapy session, before every fourth session, post-treatment, and at follow-up. Each time samples are administered early morning and late evening for 4 consecutive days [51]. Saliva samples are stored at –80° until analysis.

Hair samples are a more innovative method assumed to capture long-term systemic cortisol and consequently long-term stress, rather than momentary cortisol levels [52]. This relatively new method is thus less well validated than standard methods such as saliva samples. Samples are collected before treatment, around the 8th session, and post-treatment. A lock of about 4 mm diameter and 1–3 cm length is collected non-invasively by cutting it at the base of the vertex posterior of the head. Hair samples are stored in a dark and dry place until analysis. Cortisol concentrations in 1 cm of hair provide an indication of average cortisol level over the course of 1 month.

Blood samples are collected pre- and post-treatment. An independent lab administers them before 10 a.m. from a sober participant. Six tubes are collected: two 8-ml serum tubes, one 2-ml fluoride tube, one 2-ml EDTA tube, and one 9-ml EDTA tube. Screening of control variables is conducted within 12 hours after administration using a serum subsample, the 2-ml fluoride tube, and the 2-ml EDTA tube. The 9-ml EDTA tube is processed within 48 hours to extract DNA material, which will be stored at −80° for possible later (epi-)genetic analyses. Three other serum subsamples are stored at −80° for later analyses of biomarkers.

Mass spectometry techniques will be used for analyses of the different biological measures. For saliva samples, biomarker levels are averaged across the eight samples (4 days) to reduce daily variations.

#### Health care costs

The cost-effectiveness of therapy is assessed by means of health care cost information retrieved via the Inter-mutualistic Agency (http://www.nic-ima.be), covering a period starting 3 years pre-treatment until 2 years post-treatment (which is one of the only ways to gather objective information for a longer period before the patient entered the therapy; furthermore, it can be used to get an objective picture of antidepressant medication use). As these data are only useful when considered across a longer period and they can only be retrieved in the second wave of the study, they cannot be discussed in the primary results of the trial, but will be discussed in the second wave study on long-term outcome.

### Randomization and concealment

A (stratified) blocked randomization procedure with permuted blocks and allocation concealment to distribute dependent and self-critical patients evenly across the two types of treatment is used. An independent statistician from the Free University in Amsterdam (The Netherlands) generated the allocation sequence using the R package “Block Tools” [53]. Six trained research assistants who are blind to the allocation sequence conduct intake assessment. Only after the decision concerning eligibility is reached, will another researcher, unaware of any information on the patient, be contacted. This researcher handles the assignment to a treatment condition using the pre-generated allocation sequence. The peri- and post-interviews and follow-up assessments are conducted by the same research assistant who conducted the intake assessment, to minimize drop-out of the study. An independent researcher who is blind to the interventions and the study hypotheses conducts the primary outcome assessment (HRSD, SCID-I) immediately after treatment termination and before the post-interview (extended CCI) by the research assistant.

### Determination of sample size

Based on the literature, it can be expected that a wrong match between personality style and treatment approach can produce adverse treatment effects [4, 7, 10, 12, 13]. Therefore, we expect a large interaction effect between intervention type and personality size on the primary outcome of size 1.1 (Cohen’s *d*). To detect such an effect at the 5% significance level with at least 80% power, about 26 participants are needed in every intervention group in both strata (i.e., about 104 participants in total).

### Data analysis

Continuous variables will be summarized with means and standard deviations, median and interquartile ranges, and categorical variables with frequency tables.

The post-treatment score on the HRSD is the primary outcome. The primary hypothesis on the interaction between intervention type and personality style will be tested using a mixed model for the post-treatment score on the HRSD with baseline HRSD score (covariate), type of therapy, personality style, and the interaction of type of therapy and personality style as fixed effects and a random effect for every therapist. The latter is included to account for potential correlation in outcomes of patients assigned to the same therapist. Following the intention-to-treat approach, all randomized patients will be included in the analysis. The proposed mixed model approach is valid under the missing at random (MAR) assumption.

Secondary outcomes based on interviews (SCID and CCI) will be analyzed similarly to the primary outcome. Secondary outcomes based on self-reports (BDI, SCL, OQ, DASS, and idiosyncratic complaints), which are obtained at multiple sessions, will be analyzed using a mixed effects model approach with fixed effects of type of therapy, personality style, and its interaction at every time point; and a random effect for every therapist. The three-way interaction between therapy type, personality style, and time will be assessed to corroborate the primary hypothesis. The estimated evolution over time in each therapy group for each stratum will be graphically displayed. Effect sizes, reliable change indices (RCIs), and clinically significant change will be reported when possible for primary and secondary outcomes.

### Ethical principles

The ethical committee of Ghent University Hospital approved the entire study design and informed consent forms. Participants receive extensive written and oral explanation of all research procedures, the implications of their participation, and consent forms for both the research procedures and publishing of results. An elaborate data management plan safeguards the careful handling of confidential data in all stages of the research process.

If a serious adverse event (SAE, e.g., critical suicide risk) manifests, a Data Safety Monitoring Plan (DSMP) will be activated to decide whether or not the patient must be referred for additional interventions (e.g., antidepressant or other medication, residential care). Participants requiring mental health care in any of the follow-up measurements will be referred to adequate care.

### Data management and dissemination plan

The handling of the rich data gathered within this study is carefully planned in line with ethical considerations of privacy and confidentiality. Contact details of participants are kept in an Excel spreadsheet together with the study identifier in a separate locked location and are never used to communicate to any third party other than the researchers who require contact details to contact the participants. Quantitative data are entered into SPSS matrices throughout the study. A postdoctoral researcher conducts a biweekly control of data input. After full completion of data gathering for a participant, an additional check is performed. All data are stored on a secured university server, and back-ups are kept on encrypted USB drives. Therapists use a secured server facilitation to transfer audio files of therapy sessions to the researchers.

The features of the study and any changes to the study design are reported on Open Science Framework [54]. Quantitative data matrices without any identifying information will be made available through OSF as well. All data that imply confidentiality considerations will not be shared publicly, but are saved on the above-mentioned secured university server and can be accessed under specific conditions upon request.

This study results in a wealth of data allowing the investigation of research questions far beyond the primary hypothesis. Consequently, after the primary publication of trial results using primary and secondary outcomes, a number of studies are planned focusing, among other things, on triangulation (comparing different ways of mapping personality style), process clinical predictors (e.g., therapeutic relationship), first-person experiences (e.g., qualitative study of the CCIs), and biomarkers for depression.

## Discussion

Depressive problems are the most widely diagnosed and most widely investigated psychological problems. Numerous outcome studies have indicated the efficacy of psychotherapy and in some cases psychotherapy with medication for depressive disorders [55]. Effect sizes found in these countless randomized controlled trials comparing different interventions or interventions with no treatment are remarkably similar across studies. Moreover, the famous Dodo bird verdict, stating that there is no difference in efficacy between different types of treatment, especially holds in the context of depression [56, 57]. Nevertheless, efficacy remains limited [58], relapse rates remain rather high [59], and, most importantly, there is huge within-group variability observed in outcome research [60, 61]. Despite these important observations, little research aims at disentangling this variability within groups. In this study we want to analyze the effect of an important client variable in depression theory — namely, the personality style — and contribute to the adaptation of therapy to patient characteristics. These patient characteristics are shown to explain a large part of therapy outcomes [62, 63]; thus, the importance of exploring possibly important patient characteristics is beyond doubt. As Cuijpers [55] indicated, “more knowledge on who benefits from which treatment are important goals for future research (p. 292).”

Whether or not the primary hypothesis is confirmed, the unique properties of this study combining multiple methods and perspectives allow further in-depth exploration of processes of change, and both hindering and helpful factors in therapy. Moreover, the richness of the quantitative, qualitative, and biological data administered at multiple time points allows one to extend the primary research on group level with idiographic approaches.

Before designing the current trial, extensive piloting (naturalistic setting, 28 cases followed throughout the whole phase of therapy and follow-up) was done to test and perfect research procedures that are feasible both for participants and therapists. Also, concerning data collection and storage, the pilot study allowed us to construct a detailed manual on all the steps in the whole research process to guarantee that all research assistants can be trained and follow exactly the same procedures.

Recruitment is proceeding as planned, and several strategies (e.g., managing social media, communication with referrers) are implemented to manage recruitment in a timely manner. Recruitment is expected to last one and one-half years.

### Trial status

Recruitment is ongoing.

## Additional file

Additional file 1: SPIRIT checklist. (PDF 134 kb)

## Abbreviations

BDI-II: Beck Depression Inventory II
CBT: Cognitive behavioral therapy
CCI: Client Change Interview
CDI: Clinical Diagnostic Interview
DASS: Depression Anxiety Stress Scales
DEQ: Depressive Experiences Questionnaire
ECR: Experiences in Close Relationships questionnaire
HRSD: Hamilton Rating Scale for Depression
IIP-32: Inventory of Interpersonal Problems-32
OQ-45: Outcome Questionnaire 45
OSF: Open Science Framework
PDT: Psychodynamic therapy
PSI: Personal Style Inventory
RCI: Reliable Change Index
SCID-I: Structured Clinical Interview for DSM-IV-TR Axis I Disorders
SCID-II: Structured Clinical Interview for DSM-IV-TR Axis II Disorders
SCL-90: Symptom Checklist -90
SPIRIT: Standard Protocol Items: Recommendations for Interventional Trials
STPP: Short-term psychoanalytic psychotherapy
TRQ: Therapist Relationship Questionnaire
VAS: Visual analogue scale
VTCI: Short Temperament and Character Inventory
ZIL: Self-rating Inventory for Post Traumatic Stress Disorder
